# Distinct DNA repair pathways cause genomic instability at alternative DNA structures

**DOI:** 10.1038/s41467-019-13878-9

**Published:** 2020-01-13

**Authors:** Jennifer A. McKinney, Guliang Wang, Anirban Mukherjee, Laura Christensen, Sai H. Sankara Subramanian, Junhua Zhao, Karen M. Vasquez

**Affiliations:** Division of Pharmacology and Toxicology, College of Pharmacy, The University of Texas at Austin, Dell Pediatric Research Institute, 1400 Barbara Jordan Blvd, Austin, TX 78723 USA

**Keywords:** DNA, DNA damage and repair

## Abstract

Alternative DNA structure-forming sequences can stimulate mutagenesis and are enriched at mutation hotspots in human cancer genomes, implicating them in disease etiology. However, the mechanisms involved are not well characterized. Here, we discover that Z-DNA is mutagenic in yeast as well as human cells, and that the nucleotide excision repair complex, Rad10-Rad1(ERCC1-XPF), and the mismatch repair complex, Msh2-Msh3, are required for Z-DNA-induced genetic instability in yeast and human cells. Both ERCC1-XPF and MSH2-MSH3 bind to Z-DNA-forming sequences, though ERCC1-XPF recruitment to Z-DNA is dependent on MSH2-MSH3. Moreover, ERCC1-XPF^−^dependent DNA strand-breaks occur near the Z-DNA-forming region in human cell extracts, and we model these interactions at the sub-molecular level. We propose a relationship in which these complexes recognize and process Z-DNA in eukaryotes, representing a mechanism of Z-DNA-induced genomic instability.

## Abstract (web summary)

Z-DNA-forming CG repeats are mutagenic in mammalian cells but the mechanism has remained unknown so far. Here, the authors show that the nucleotide excision repair complex Rad10-Rad1 (ERCC1-XPF) and the mismatch repair complex Msh2-Msh3 (MSH2-MSH3) are required for Z-DNA-induced genetic instability in yeast and human cells.

## Introduction

**Fig. 1:**
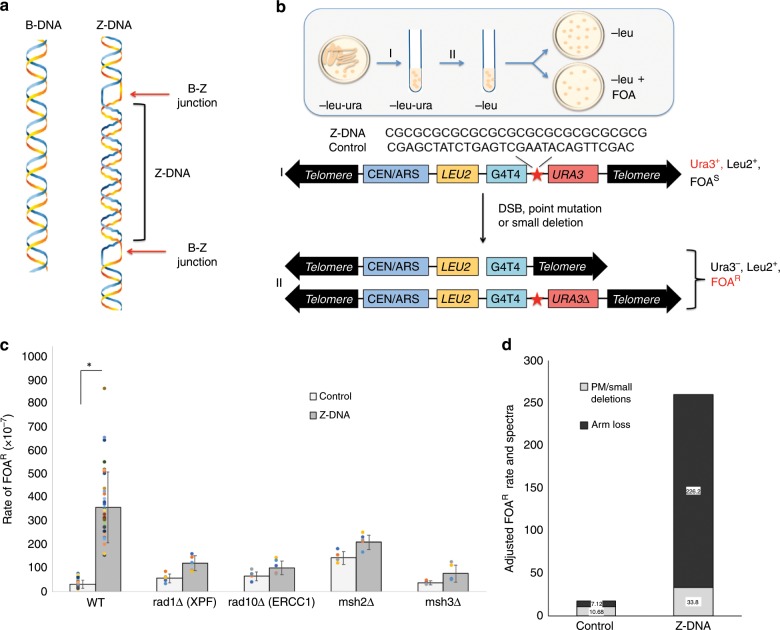
Rad10-Rad1 and Msh2-Msh3 are required for Z-DNA-induced genetic instability in *S. cerevisiae*. **a** B-DNA (left panel) and left-handed Z-DNA flanked by two regions of B-DNA (right panel) B-Z junctions are labeled (red arrows). **b** Schematic of the YAC fragility assay. **c** Mutation rates calculated as rate of FOA^R^ for WT, *rad1Δ*, *rad10Δ*, *msh2Δ*, and *msh3Δ* strains containing control B-DNA or Z-DNA-forming YACs [Student’s *t*-test was used to calculate a *P* value. *P < 0.001]. Data are presented as means ± SEM of triplicate experiments, and values of each repeat are shown as dot plots on the bars. **d** Mutation spectra of FOA^R^ clones from control B-DNA or Z-DNA-forming YACs in the WT strain were determined by sequencing and PCR, and mutants were categorized as either point mutation (PM)/small deletion within the *URA3* gene or complete loss of the right arm of the chromosome as a result of a DSB^60^. “Adjusted FOA^R^ rate and spectra” was calculated by multiplying the percentage of each category (calculated from 30 mutants) by the total FOA^R^ ratio. See also Supplementary Figs. 1 and 2, and Supplementary Tables 1 and 2.

Since the discovery of the B-DNA double helix, several alternative DNA structures (i.e., non-B DNA) have been described, including Z-DNA, which has been studied extensively by our laboratory^1–4^ and others^5–7^. Z-DNA is a left-handed helix that forms at sequences of alternating purine-pyrimidine repeats (e.g., TG or GC) (Fig. 1a), named after its zigzag arrangement of the sugar-phosphate backbone, as the purines in Z-DNA are inverted into the *syn* conformation, while the pyrimidines remain in the *anti* conformation^8^. Sequences with the capacity to adopt Z-DNA structures are abundant in the human genome, occurring ~1/3000 bp^9^.

The biological function of Z-DNA has been of interest due to the high occurrence and conservation of Z-DNA-forming sequences across multiple species of eukaryotes^10^, and the existence of a class of specific Z-DNA-binding proteins in eukaryotic cells that play important roles in transcription, recombination, RNA editing, viral pathogenicity, tumor development, and evolution^11–14^. Although the function of Z-DNA remains to be fully understood, many studies have revealed a number of potential roles for the structure in DNA transactions including DNA replication and gene expression [reviewed in^15^]. Of particular interest, non-B DNA-forming sequences can also contribute to genomic instability and evolution^10,14^. For example, Z-DNA-forming sequences are significantly enriched at sites within or near chromosomal translocation breakpoint hotspots in human cancer genomes^4^. Moreover, we have discovered that Z-DNA-forming CG repeats are mutagenic in mammalian cells and in genomes of transgenic mice, and that these sequences can stimulate the formation of DNA double-strand breaks (DSBs) resulting in large deletions^1,3,16^, implicating them in cancer etiology. The genetic instability seen in mammalian cells appears to be unique to the Z-DNA structure and not just the purine-pyrimidine repeats, because dinucleotide AT repeats of the same length (which cannot form Z-DNA) are much more stable, and the resulting mutants are predominantly small indels that change the repeat numbers^1,17^. However, the molecular mechanisms involved in Z-DNA-induced genomic instability and the generation of DSBs are not known.

Here we show that the Rad10-Rad1 (ERCC1-XPF) complex that acts in the nucleotide excision repair (NER) pathway, and the Msh2-Msh3 (MSH2-MSH3) complex that acts in the mismatch repair (MMR) pathway, interact on Z-DNA and are required for Z-DNA-induced genetic instability in yeast and human cells. Interestingly, the interactions of these proteins on Z-DNA appear to be outside of their canonical roles in the NER and MMR mechanisms, given that not all NER or MMR proteins were required for Z-DNA-induced genetic instability in this study. Thus, we propose a relationship between the ERCC1-XPF and MSH2-MSH3 complexes in recognizing and processing Z-DNA that is unique and distinct from their roles in canonical DNA repair pathways. This relationship represents a mechanism of genomic instability, further implicating Z-DNA in translocation-related diseases and cancer etiology.

## Results

### Proteins involved in Z-DNA-induced mutagenesis in yeast

We chose a Z-DNA-forming CG14 repeat in this study, as we have demonstrated that it forms a Z-DNA structure that stimulates the formation of DSBs and large deletions in mammalian cells, rather than forming small loops that can occur at simple repeats^1^. Yeast provide a useful eukaryotic system for studying mechanisms involved in genetic instability since mutant libraries deficient in non-essential genes are commercially available [http://www-sequence.stanford.edu/group/yeast_deletion_project/deletions3.html]. To determine the mutagenic potential of Z-DNA-forming sequences on yeast chromosomes, we used a yeast artificial chromosome (YAC) fragility assay, which utilizes the *URA3* gene as a reporter. Either a Z-DNA-forming sequence (CG14) or a control B-DNA-forming sequence was inserted into the YAC adjacent to the *URA3* gene^18,19^, where the structure-induced chromosomal breakage results in loss of *URA3*, as assessed by 5-Fluoroorotic acid resistance (FOA^R^) (Fig. 1b). We found that the 28-bp Z-DNA-forming sequence stimulated mutations ~11.3-fold over that of the control B-DNA-forming sequence in the wild-type yeast cells (Fig. 1c) in the absence of exogenous DNA-damaging factors.

FOA^R^ colonies could arise from different mechanisms; DSBs that occur near the Z-DNA-forming sequence resulting in the loss of the right arm of the YAC, or other events such as point mutations or deletions within *URA3* (Fig. 1b). To determine if FOA^R^ was due to complete loss of the right arm of the YAC, or due to inactivation of the *URA3* gene by a point mutation/small deletion, we analyzed FOA^R^ colonies by PCR using primers that amplify the Z-DNA or control B-DNA sequence, a region of the *URA3* gene, or a region of the *LEU2* gene (Supplementary Fig. 1, and Supplementary Table 1). In the WT strain, the majority of the Z-DNA-induced mutants resulted from the loss of the right arm of the YAC, likely due to DSBs (indicated by negative PCR results from primers that amplify the Z-DNA or control B-DNA sequence), while the majority of the spontaneous background mutants on the control YAC were likely point mutations/small deletions (indicated by intact PCR products). Figure 1d shows the adjusted FOA^R^ rate and spectra of Z-DNA or control B-DNA in the WT stain, calculated by multiplying the percentage of each category (from 30 mutants characterized) by the total FOA^R^ rate of control B-DNA or Z-DNA. The vast majority of the genetic instability events induced by Z-DNA were arm loss, suggesting a unique mechanism of Z-DNA-induced genetic instability, which involves the generation of DSBs.

To identify gene products involved in Z-DNA-induced genetic instability in yeast, we transferred the YACs containing a control B-DNA or a Z-DNA-forming sequence to mutant yeast strains from an *S. cerevisiae* genome library via *kar*-crossing^18,20,21^, and measured Z-DNA-induced loss of *URA3* in the various mutants. As expected, deficiencies of many DNA repair proteins increased the background spontaneous arm loss events on the control B-DNA-containing YAC (Fig. 1c). However, among the 15 different repair genes tested (see Supplementary Table 2), the most striking variations in Z-DNA-induced chromosome arm loss resulted from gene deficiency in the NER mutants *rad1Δ(XPF)* and *rad10Δ(ERCC1)* (where instability was decreased from 11.3-fold above the control YAC in WT to 2.1-fold and 1.6-fold in the mutants, respectively), and MMR mutants *msh2Δ* and *msh3Δ* (from 11.3-fold above the control YAC in WT to 1.5-fold and 2.0-fold in the mutants, respectively) (Fig. 1c), suggesting that these proteins are required for Z-DNA-induced genetic instability. Surprisingly, the dramatic effects seen in these mutants were not observed in other mutants within the same DNA repair pathways, i.e., rad4 (XPC), rad14 (XPA), rad2 (XPG), and rad26 (CSB) in the NER pathway, and msh6, mlh1, and exo1 in the MMR pathway (Supplementary Fig. 2). These results suggested that the Rad10-Rad1(ERCC1-XPF) and Msh2-Msh3 complexes, rather than the entire functional NER and/or MMR pathways, were involved in Z-DNA-induced genetic instability. Thus, these findings suggest a mechanistic pathway for the mutagenic processing of Z-DNA in eukaryotic cells.

Furthermore, Z-DNA-induced mutation spectra were dramatically altered in the NER-deficient and MMR-deficient strains (Supplementary Table 2). In *Δrad10(ERCC1)*, *Δrad1(XPF), Δmsh2*, and *Δmsh3* strains where Z-DNA-induced instabilities were significantly reduced, Z-DNA-induced mostly point mutations rather than large deletions. Thus, deficiency of these genes specifically diminished larger-scale chromosome arm loss. Clearly, these repair proteins were required for Z-DNA-induced chromosomal breakage in yeast, while other proteins within the NER or MMR pathways were not directly involved in this process.

### Z-DNA-induced genetic instability in human cells

**Fig. 2:**
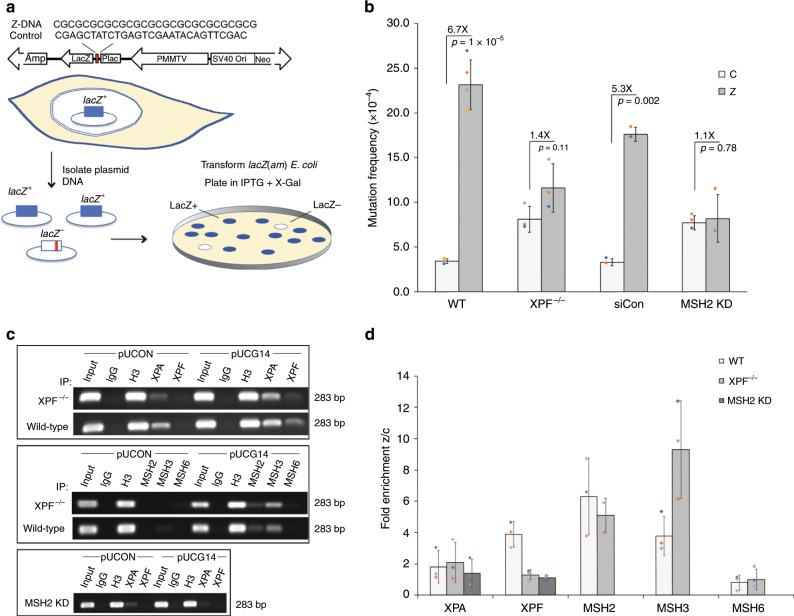
ERCC1-XPF and MSH2-MSH3 are required for Z-DNA-induced mutagenesis in human cells. **a** Schematic of blue-white mutation assay (adapted from Vasquez et al.^61^). **b** Mutation frequencies for WT, XPF-deficient, siRNA control, and siRNA-depleted MSH2 human cell lines. Student’s *t*-test was used to calculate statistical values. **c** Representative ChIP analysis performed on (pUCON and pUCG14) plasmids in WT and XPF-deficient human cells using antibodies against NER (top panel) and MMR (middle panel) proteins, and in MSH2-depleted cells (bottom panel). IgG and H3 served as negative and positive controls, respectively. **d** Quantification of ChIP analysis comparing the Z-DNA-forming sequence to the control B-DNA region (Z/C), normalized to input. Data in **b**, **d** are presented as means ± SEM of triplicate experiments, and values of each repeat are shown as dot plots on the bars. See also Supplementary Figs. 3–7, and Supplementary Table 1.

To determine if the results we found in yeast translated to higher eukaryotes, we screened the same genes to determine if the ERCC1-XPF and MSH2-MSH3 repair complexes were required for Z-DNA-induced genetic instability in human cells. We performed mutagenesis assays via blue-white screening^1,19^ in repair-deficient cells and control WT cells (Fig. 2a). Isogenic human XPF-proficient or XPF-deficient cells (Supplementary Fig. 3a) were transfected with mutation-reporter plasmids containing either a control B-DNA-forming or a Z-DNA-forming sequence (Fig. 2a). We found that in WT human cells, Z-DNA resulted in a significant, 6.7-fold increase in mutations compared to the control reporter plasmid (Fig. 2b, *P* value = 1 × 10^−5^), consistent with our previous reports^1^. In contrast, we found that in the XPF-deficient human cells, the Z-DNA-induced mutation frequency was diminished to the control level (~1.4-fold of that of the control B-DNA sequence, *P* value = 0.11, Fig. 2b, vs. 6.7-fold of that of control in the XPF-proficient WT human cells, *P* value = 0.00005). Although the majority (>95%) of the mutants characterized from both the control B-DNA or Z-DNA in both cell lines were deletion events, the dramatically reduced mutation frequency induced by Z-DNA in XPF-deficient human cells suggested that the ERCC1-XPF repair complex was required for Z-DNA-induced genetic instability in human cells. Additionally, we performed the mutagenesis assay in isogenic human XPA-proficient or XPA-deficient cells and found that the absence of XPA had a very minor effect on Z-DNA-induced mutagenesis (Supplementary Fig. 4). These results are in accordance with our yeast data, and suggested that ERCC1-XPF was acting outside NER in the mechanism responsible for Z-DNA-induced genomic instability. Interestingly, these results differ from our recent findings demonstrating that the canonical NER mechanism is required for the mutagenic processing of another alternative DNA structure, H-DNA^22^, suggesting that different DNA structures are processed by different mechanisms.

Similarly, we determined the extent to which the MSH2-MSH3 complex was required for Z-DNA-induced genetic instability in human cells. Using targeted siRNA knockdown, we depleted MSH2 from the WT cells (by ~80–95% of endogenous levels; Supplementary Fig. 3b). Depletion of MSH2 in the WT cells also diminished Z-DNA-induced mutagenesis, resulting in 1.1-fold over the control B-DNA sequence (Fig. 2b, *P* value = 0.78). In contrast, following treatment with a non-targeting siRNA, the Z-DNA-induced mutagenesis still remained high, and was ~ 5-fold higher than the control (Fig. 2b, *P value* = 0.002), similar to the induction seen in WT cells. As expected, higher percentages of point mutations were observed from both control and Z-DNA plasmids in MSH2-deficient cells, due to the lack of MMR function. The significantly reduced Z-DNA-induced mutation in MSH2-deficient cells suggested that MSH2 was also required for Z-DNA-induced genetic instability in human cells, consistent with our data from yeast (Fig. 1c).

Given that MSH2-MSH3 can bind to small loops/bubbles that can form on simple repetitive sequences, MSH2 deficiency could potentially affect genetic instability within the CG-repeats without the involvement of a Z-DNA structure. Therefore, we also included several other repeats in our study, such as AT14^1^ and GT14 (Supplementary Fig. 5a). AT14 repeats can form hairpins or loops^1,17^, and GT14 repeats can form small loops, but neither can form Z-DNA (GT requires ~30 repeats to form Z-DNA)^17,23^. Both the AT14 and GT14 repeats were much more stable in mammalian cells than the Z-DNA-forming CG14 repeat (Supplementary Fig. 5b and Ref. ^1^). In a separate study we found that longer GT30 and GT41 repeats that readily form Z-DNA, were mutagenic in both mammalian cells and in yeast cells. They also appeared to be related to genetic instability and evolution in stickleback fish^14^. To exclude the possibility that the instability was caused simply by the CG content in CG14, a CCGCGG5 repeat, containing a similar CG content, but unable to adopt a Z-DNA structure (Supplementary Table 1) was also included and we found that the mutation frequency induced by the CCGCGG(5) repeat was not significantly different than the control B-DNA sequence (*P* value = 0.32). The mutants were predominantly deletions of the repeat unit, and not the large-scale deletions that result from the Z-DNA-forming CG14 repeat (Supplementary Fig. 6). Although the CCGCGG(5) repeat is a simple repeat and has the potential to form cruciform structures, the kinetics of the formation of cruciforms are slow at physical conditions^24,25^. These data suggested that the Z-DNA structure formed at the CG14 sequence had a unique mechanism of mutagenic processing in mammalian cells.

### NER and MMR proteins associate with Z-DNA-forming sequences

To further investigate the mechanistic roles of the ERCC1-XPF and MSH2-MSH3 repair complexes in Z-DNA-induced genetic instability, we determined whether these complexes interacted with the Z-DNA-forming sequence in human cells. We performed chromatin immunoprecipitation (ChIP) assays in the WT and repair-deficient human cell lines that were used for the mutagenesis studies. Using antibodies against NER proteins (XPA and XPF) and MMR proteins (MSH2, MSH3, MSH6), our results in WT cells revealed an ~4-fold enrichment of XPF at the Z-DNA-forming region compared to the control B-DNA region (Fig. 2c top panel, and Fig. 2d), and an ~6-fold, and ~4-fold enrichment of MSH2 and MSH3, respectively (Fig. 2c middle panel, and Fig. 2d). These results suggested that these proteins associated selectively with Z-DNA, and perhaps played roles in the recognition and/or processing of the structure-forming sequence.

To determine if the interactions of ERCC1-XPF and MSH2-MSH3 on Z-DNA were co-dependent, we determined the enrichment of MSH2-MSH3 at Z-DNA in XPF-proficient and deficient human cells, and conversely, the enrichment of ERCC1-XPF at Z-DNA in MSH2-proficient or depleted human cell lines. Our results indicated that the recruitment of MSH2-MSH3 to Z-DNA was independent of XPF, as the complex was enriched at the Z-DNA-forming site in both XPF-proficient and XPF-deficient human cell lines. Surprisingly, MSH2 depletion diminished the enrichment of XPF at the Z-DNA-containing region detected in WT (XPF-proficient) cells (4-fold enrichment of Z/C in wild-type decreased to 1.1-fold in MSH2-depleted cells) (Fig. 2c bottom panel, and Fig. 2d). We repeated the experiment in MSH2-deficient human Hec59 cells and the isogenic ch2 add-back MSH2-proficient Hec59 + ch2 cells and quantified the enrichment of XPF at Z-DNA-forming and control regions via real-time qPCR (see Supplementary Methods). The results revealed an ~3-fold decrease in XPF at the Z-DNA region compared to the control region in MSH2-deficient cells over that in the MSH2-proficient cells (Supplementary Fig. 7). These results suggested that MSH2-MSH3 bound the Z-DNA region and recruited the ERCC1-XPF complex to the site.

In addition, we determined the interactions of additional NER and MMR proteins at the Z-DNA-forming region, and found that XPA also associated with the Z-DNA-forming region, but was only slightly enriched (~1.8-fold) over its association with the control B-DNA region, regardless the status of XPF (Fig. 2c, d). This result suggested that the interaction of XPA with Z-DNA was either less specific and/or more transient than that of XPF in human cells. In contrast to our results with XPF, there was no effect on XPA recruitment by MSH2 depletion in human cells, also suggesting that XPA does not appear to be necessary for the enrichment of XPF at the Z-DNA region in our system. MSH6 exhibited no detectable enrichment in the presence or absence of XPF (Fig. 2c, d). Taken together, these data suggest that the interaction between the ERCC1-XPF and MSH2-MSH3 complexes plays a unique, functional role in Z-DNA-induced genetic instability, in a mechanism distinct from that of their roles in the NER and MMR pathways.

### In vitro cleavage of Z-DNA by ERCC1-XPF

**Fig. 3:**
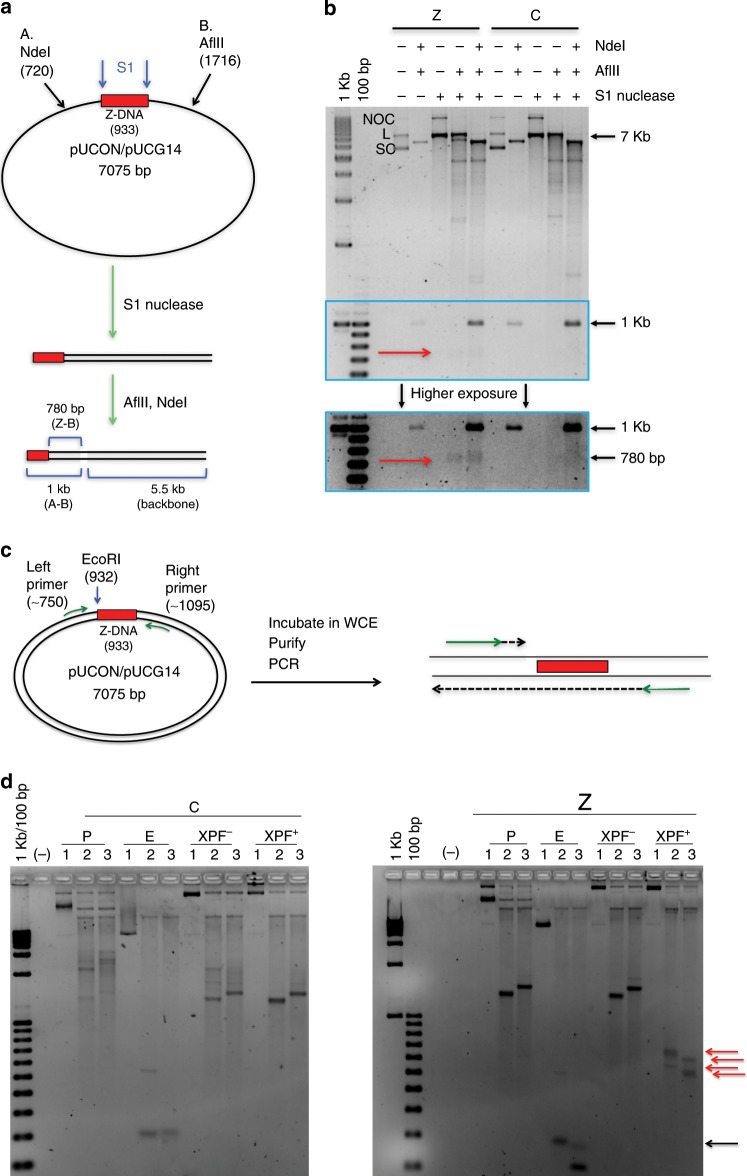
ERCC1-XPF cleaves near the Z-DNA-forming region. **a** S1 nuclease assay schematic. **b** Top panel: SYBR Gold-stained agarose gel demonstrating S1 cleavage at single-stranded regions corresponding to the B-Z junctions on the Z-DNA-forming plasmid (Z), resulting in ~780 bp fragment (red arrow) that is absent in the control B-DNA plasmid (C). Bottom panel: higher exposure of S1 cleavage product. [NOC, nicked open circular; L, linear; SC, supercoiled species]. **c** PCR primer extension assay schematic using plasmid DNA in human XPF-proficient or XPF-deficient WCE. Purified DNA was used as a template for a primer extension assay using either the left or right primer (green arrows). **d** PCR products were separated on a 1.5% agarose gel revealing extra cleavage products (red arrows) from the Z-DNA plasmid in XPF-proficient WCE. Plasmid DNA only (P) served as a negative control, and EcoRI (E), restriction at the Z-DNA-forming insert served as a positive control, resulting in ~160 or ~180 bp products (black arrow). Lane 1: template only; lane 2: left primer; and lane 3: right primer. See also Supplementary Table 1. Extension products of >1000 bp resulted from primer “run off” from the template. Shorter extension products resulting from cleavage on plasmids in WCE are indicated by red arrows.

Previously, we have shown that Z-DNA-forming sequences can stimulate the formation of DSBs, resulting in large-scale deletions^1^, but the nucleases that cleave the Z-DNA-forming sequences were unknown. Here, we found that the structure-specific nuclease, ERCC1-XPF bound to Z-DNA and was required for the mutagenic processing of the Z-DNA-forming sequences. Thus, we determined whether ERCC1-XPF processes this structure resulting in DNA strand breaks by using an in vitro cleavage assay on Z-DNA-forming sequences on supercoiled plasmid DNA. We used single-strand-specific S1 nuclease to confirm the presence of single-stranded regions at the B-Z junctions^1^ (Fig. 3a). Under Z-DNA-forming conditions, S1 nuclease cleaved DNA at the Z-DNA-forming sequence resulting in an ~780 bp fragment, indicating the formation of a B-Z junction, which was not present following S1 digestion of the control B-DNA-forming plasmid (Fig. 3b). Next, we performed an in vitro cleavage assay using human whole cell extract coupled with a primer extension assay to detect DNA cleavage on either side of the Z-DNA. The control (C) or Z-DNA-forming (Z) plasmids were incubated with whole cell extract^26^ prepared from the same human XPF-proficient (XPF^+^) and XPF-deficient (XPF^-^) cell lines used in the mutagenesis and ChIP assays. By extending a primer bound upstream or downstream of the Z-DNA-forming region, we detected a long “run-off” product >1000 bp on plasmid only (P) and plasmids incubated in XPF + and XPF- WCEs (Fig. 3c). An EcoRI digested plasmid that was cut immediately next to the CG14 repeat served as a positive control for cleavage near Z-DNA. Some primer extension was terminated earlier on the Z-DNA plasmid incubated with XPF + WCE, resulting in shorter extension products, and such shorter extension products were not present in the XPF- WCE, nor in the control B-DNA-containing region in XPF + or XPF- WCE (Fig. 3d, red arrows). Interestingly, shorter products were detected with both the left and right primers, suggesting that ERCC1-XPF can cleave both upstream and downstream of the Z-DNA-forming sequence, further implicating this repair complex in Z-DNA-induced genetic instability in eukaryotes.

### Simulation of ERCC1-XPF and MSH2-MSH3 interactions with Z-DNA

We simulated the human ERCC1-XPF and MSH2-MSH3 complexes bound to a Z-DNA structure to ascertain more detailed insight into their interactions at a sub-molecular level. In silico computational studies were used to model the ERCC1-XPF complex, which was constructed using Swiss-Model [https://swissmodel.expasy.org]. XPF [https://www.uniprot.org/uniprot/Q92889] was modeled with the XPF HhH domains (http://www.rcsb.org, 10.2210/pdb1z00/pdb] and the nuclease domain of human XPF bound to dsDNA as a template [http://www.rcsb.org, 10.2210/pdb2bgw/pdb], and the DNA excision repair protein ERCC1 was modeled [https://www.uniprot.org/uniprot/P07992] with the ERCC1 central domain as the template [http://www.rcsb.org, 10.2210/pdb2jpd/pdb]. A model of the MSH2-MSH3 complex was generated using the program PyMol [http://www.pymol.org], based on the crystal structure of the MSH2 and MSH3 proteins [http://www.rcsb.org, 10.2210/pdb3thy/pdb].

**Fig. 4:**
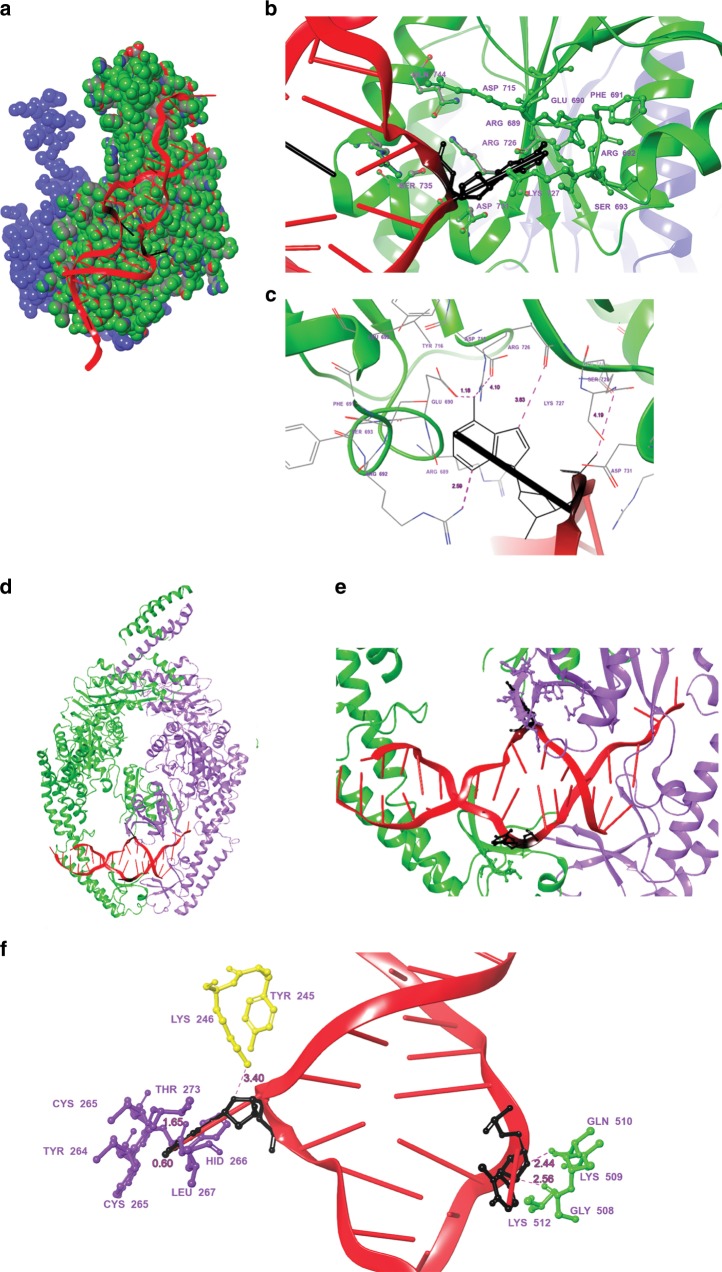
Models of the ERCC1-XPF and MSH2–3 complexes docked to Z-DNA. **a** Corey-Pauling-Koltun (CPK) sphere representation of the ERCC1-XPF complex (ERCC1 complex in blue and XPF in green) docked to Z-DNA (red, B-Z junction in black) at the site with maximum interaction with the protein. **b** Interacting interface residues between the B-Z junction nucleotides with the ERCC1-XPF complex. **c** Distances between the major interacting and active residues with the B-Z junction. **d** Model showing different positions of Z-DNA (red, PDB: 2ACJ) bound to the MSH2-MSH3 complex (MSH2: green, MSH3: cyan, PDB: 3THY) in the increasing order of their interactions with the binding site residues (from left to right). **e** Highlighted interaction (from “**d**”) between the B-Z junction and the binding residues within 3 Å of the nucleotides. **f** Distances and labels of the interacting residues with the B-Z junction are highlighted.

The potential interactions of the ERCC1-XPF and MSH2-MSH3 complexes with Z-DNA were predicted using both NPDock and HDock. NP-dock’s fully automated procedure can predict accurate and unbiased interactions, but the molecules to be modeled are limited to a maximum of 10,000 atoms; while HDock does not have this limitation, it requires users to provide plausible binding information. Thus, combining the data from both approaches can reduce these limitations. Figure 4 shows the predicted models generated from the data that were most consistent between NPDock and HDock. The predicted models showed interactions of the ERCC1-XPF and MSH2-MSH3 complexes with the B-Z junction, thus providing evidence for their interactions with Z-DNA (Fig. 4). The site of maximum interaction with Z-DNA was seen at the XPF endonuclease domain (673–737 residue-containing region) of the ERCC1-XPF complex (Fig. 4a, b). Interface residues are highlighted to depict the number of significant residues that are potentially involved in binding/recognition of Z-DNA (Fig. 4b). We then measured the distances between the B-Z junction nucleotides to the interacting residues of the XPF endonuclease domain that were under 5 Å (Fig. 4c). This model predicts the most probable interaction sites between the ERCC1-XPF complex and Z-DNA, whereby the B-Z junction was found to interact with Asp715, Tyr 716, Arg 726, Lys727, Ser 728, Asp 730 etc., which are essential for the catalytic activity of the XPF endonuclease domain^27^. This compares well with the previously described model of ERCC1-XPF binding to a splayed-arm DNA substrate, which also positioned the junction near the XPF nuclease domain^28^.

Similarly, the interactions occurring between the B-Z junction and the MSH2-MSH3 complex were modeled by both NPdock and HDock. As there were multiple sites of interaction, the most probable sites were sorted based on their binding affinities with interacting partners. The most plausible models that were consistent between the two tools are shown in Fig. 4d–f, where the B-Z junction was located between the mismatch binding domain (MBD) and the clamp domains of the MSH2-MSH3 complex, and one of the single strands at the B-Z junction that interacted with the MBD (Fig. 4d). This is very similar to previously reported models of the MSH2-MSH3 complex bound to a G:T mismatch^29^. The major interacting residues with the B-Z junction within 3 Å are highlighted to depict the number of significant residues that are potentially involved in binding/recognition of Z-DNA (Fig. 4e). Of note, we found that the active residues Tyr245 and Lys246 that are known to interact with unpaired loops^30,31^ were positioned very near the B-Z junction. The distances between the B-Z junction nucleotides to the labeled residues are listed in Fig. 4f. This model predicts the most probable interaction sites between MSH2-MSH3 and Z-DNA.

## Discussion

We previously found that Z-DNA-forming sequences are mutagenic and stimulate DSBs, resulting in deletion events in mammals^1,3,16^. Here, we discovered that a Z-DNA-forming sequence also stimulates DSBs and genetic instability in yeast cells. These results suggest that eukaryotic cells may share a common and conservative mechanism in generating mutations at Z-DNA-forming sequences.

To understand the mechanism(s) involved, we screened a yeast mutant library to identify gene products that are involved in Z-DNA-induced genetic instability using a YAC reporter system, and found that several DNA repair proteins are involved. Among these genes, we found that Rad10-Rad1 or Msh2-Msh3 deficiency in yeast (ERCC1-XPF or MSH2-MSH3 in humans) dramatically reduced Z-DNA-induced chromosome breakage near the Z-DNA-forming region. These results are consistent with our recent publications that DNA repair proteins are involved in processing non-B DNA structures in a mutagenic fashion, including H-DNA and cruciform structures, supporting our model of “structure-specific cleavage” by DNA repair proteins in non-B DNA-induced genetic instability^22,32^. Here we found that Z-DNA-forming sequences are processed by a mechanism involving proteins from both the NER and MMR mechanisms. This differs from our recent findings on another non-B DNA structure, H-DNA, that is processed by the canonical NER pathway^22^. NER and MMR pathways recognize distortions and mismatches/bubbles on DNA and cleave DNA as part of “repair” processing. However, these DNA strand breaks could lead to chromosome breakage and other mutagenic events if repair is not completed faithfully.

Interestingly, many other repair proteins required in the NER or MMR mechanisms are not involved in Z-DNA-induced genetic instability, suggesting that ERCC1-XPF and MSH2-MSH3 process Z-DNA in a mechanism outside of their canonical roles in the NER and MMR pathways. We confirmed our results from yeast in human cells, and demonstrated that both ERCC1-XPF and MSH2-MSH3 complexes, but not other repair proteins in the NER or MMR mechanisms, were enriched at Z-DNA-containing regions relative to control B-DNA regions. To rule out the possibility that an R-loop forms at the repetitive area during transcription, which in turn may recruit the MSH2-MSH3 and ERCC1-XPF complexes, we performed ChIP assays and found no evidence of R-loop formation at the CG repeat (Supplementary Fig. 8). These results suggest that the activities of ERCC1-XPF and MSH2-MSH3 in Z-DNA-induced genetic instability may be a mechanism of processing Z-DNA specifically common in eukaryotes.

Further, we verified the cleavage activity of ERCC1-XPF near the Z-DNA sequence in human cell extracts and identified incisions on both sides of the Z-DNA-containing region in XPF-proficient WCE. These incisions were diminished in XPF-deficient WCE, further supporting our model of “structure-specific cleavage” of non-B DNA-forming sequences in eukaryotes. In the NER mechanism, ERCC1-XPF is recruited to the 5′ side of the damage and incision occurs on the damaged strand of the duplex ~2–8 nucleotides away from the double-strand/single-strand DNA (ds/ssDNA) junction generated around the damaged site^33^. However, in this assay the cleavage sites were identified up to hundreds of bp away from the ds/ssDNA junction (i.e. the B-Z junction), consistent with our previous studies that Z-DNA can stimulate the formation of multiple DSBs in regions up to hundreds of bps upstream and downstream of the CG repeat^1^. Since the reaction was performed in cell extracts, it is possible that the initial cleavage sites occurred near the Z-DNA region, but the breakpoints were further processed for DSB repair.

Further, we demonstrated that binding of ERCC1-XPF at the Z-DNA region was dependent on the MSH2-MSH3 complex. In both siRNA-depletion experiments and MSH2-deficient human cells, enrichment of XPF on Z-DNA was dramatically reduced (Fig. 2, Supplementary Fig. 7). A physical interaction between Msh2(MSH2) and Rad1(XPF) and Rad10(ERCC1) has been shown in yeast by two-hybrid analysis^34^ and in mammalian cells^35^; the two complexes can work together in processing recombination intermediates for efficient targeted gene replacement and single-strand annealing events, in which Msh2-Msh3 is thought to either recruit and/or aid in the cleavage activity of Rad10-Rad1^36,37^. Msh2-Msh3 and Rad10-Rad1 are also known to cooperate in the resolution of mismatches that can form between heteroalleles during meiotic recombination^38,39^. In mammalian cells, the two complexes have been shown to cooperate in a mechanism responsible for cellular resistance to chemotherapeutic DNA interstrand crosslinking (ICL) agents^35^, and we have previously shown that the MMR proteins, MSH2-MSH3 and MLH1, are required for the repair of triplex-forming oligonucleotide (TFO)-directed psoralen ICLs^40^, and that MMR and NER proteins can interact in recognizing TFO-directed ICLs^41^. Importantly, this finding indicates that these repair complexes work together to process alternative DNA structures (e.g., Z-DNA) in the absence of DNA damage per se.

Interestingly, the ERCC1-XPF and MSH2-MSH3 complexes are also involved in the 3′ non-homologous tail removal (3′NHTR) mechanism in DSB repair, a necessary process to remove flapped strands 3′ to the homologous sequences used for single-strand annealing or gene conversion^42–44^. DNA polymerase and ligase fill and seal the gap only after the unannealed 3′-tails are removed. Eichmiller et al. (2018) reported that the Msh2-Msh3 complex could bind to the 3′-free tail and then recruit Rad1–10 and Saw1 in yeast^45^. Saw1 can directly stimulate the endonuclease activity of Rad1–10^44^ in processing the 3′-tails.

To determine whether ERCC1-XPF and MSH2-MSH3 were recruited to the CG-repeat region to process the Z-DNA structure, resulting in DNA breakage, and/or if they were involved in end processing via 3′NHTR with Saw1, we determined the Z-DNA-induced YAC fragility in Saw1-deficient yeast compared to WT cells. Although Saw1 deficiency reduced fragility of both the control B-DNA and the Z-DNA-containing YACs, the ratio of Z-DNA-induced fragility over control B-DNA was similar (~5 fold) in both WT and Saw1-deficienct cells (Supplementary Fig. 9), demonstrating that Saw1 was not required for Z-DNA-induced mutation. Since Saw1 is essential for Rad1 recruitment and cleavage at 3′ tailed recombination intermediates and 3′NHTR^40,45^, this result suggested that the Rad1–10 complex was involved in Z-DNA processing in a pathway unique to that of 3′NHTR. Furthermore, in the ChIP assays the primers were designed to amplify a region containing the Z-DNA-forming sequence so that a positive PCR amplification suggested that at least some of the ERCC1-XPF and MSH2-MSH3 complexes bound on the intact DNA, and not on 3′-tails generated from nicked DNA or DSBs. It is worth noting that Rad1–10, Msh2-Msh3, and the MRX complexes are also involved in short-sequence recombination in yeast^46,47^, so these proteins can contribute to the genetic instability via multiple mechanisms.

The interactions of Z-DNA with the DNA repair protein complexes, ERCC1-XPF and MSH2-MSH3, were demonstrated at the sub-molecular level by in silico modeling and docking studies (Fig. 4). In these models, the B-Z junctions were located near the active sites of the enzymes (Fig. 4), similar to previously reported models with branched structures or mismatched substrates^31,48–50^, and supporting the cleavage activity that we found by ERCC1-XPF on Z-DNA (Fig. 3).

**Fig. 5:**
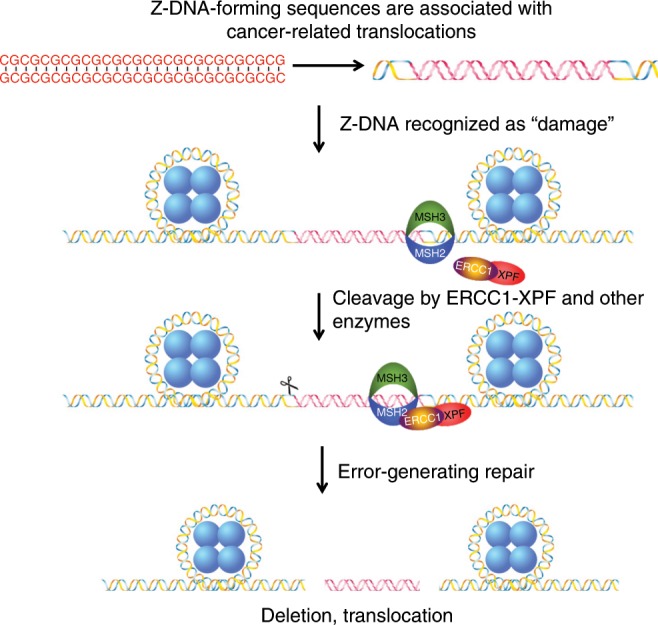
Proposed mechanism for Z-DNA-induced genetic instability in eukaryotes. During DNA metabolic processes, the CG repeat sequence is unwrapped from histones (blue circles) and negative supercoiling is generated, which stimulates Z-DNA formation. The structure of Z-DNA is recognized as “damage” by the MSH2-MSH3 complex, signaling repair. The ERCC1-XPF complex is recruited to the site for cleavage near the Z-DNA-forming region (processing of the B-Z junction on the right side of Z-DNA is shown in the figure; however, similar processing could also occur at the other B-Z junction on the left, marked as a scissor), resulting in DSBs in an attempt to repair the “damage”. The breaks may be processed in an error-free fashion, or the breaks may be processed in an error-generating fashion resulting in genomic instability in the form of large deletions and translocations, which may contribute to disease etiology.

Collectively, the data obtained in this study indicate distinct functions for the MSH2-MSH3 and ERCC1-XPF repair complexes in processing Z-DNA, which involves cross-talk between these proteins outside of canonical repair pathways. We propose a model (Fig. 5) in which Z-DNA is recognized by MSH2-MSH3 as “damage” and recruits the structure-specific nuclease, ERCC1-XPF, which then processes the alternative DNA structure in an attempt to remove the “damage”. This processing can lead to DSBs, potentially resulting in large deletions or translocations at or near the site of the Z-DNA-forming sequence, providing a potential explanation for the co-localization of translocation hotspots with Z-DNA-forming sequences in human cancer genomes^4,7^.

While we have identified the MSH2-MSH3 and ERCC1-XPF complexes to be involved in Z-DNA-induced genetic instability in this study, it is likely that other proteins are also involved in this mechanism and future studies are warranted to identify potential candidates, which will provide insight into the contribution of Z-DNA-induced genomic instability to the development of human disease. Understanding the mechanisms involved in DNA structure-induced genetic instability in disease etiology will allow for the advancement of therapeutics to treat and/or prevent diseases of genetic instability, such as cancer.

## Methods

### Yeast strains and yeast artificial chromosomes (YACs)

The yeast strain 213 cir (*MATa Kar1-1 leu2-3, 112 ura3-52 his7 cyh2*^*R*^*)* was used for YAC transfer into other yeast strains. The yeast strain BY4742 (*MATα, his3Δ1, leu2Δ0, lys2Δ0, ura3Δ0*) and derivatives (Yeast deletion library GSA-5, ATCC, Manassas, VA) were used in DNA structure-induced YAC fragility and spectra assays. We used the YAC system as described by Freudenreich’s group^18^, containing either a Z-DNA-forming or control B-DNA-forming sequence (Supplementary Table 1), and functional *LEU2* and *URA3* genes were transferred from donor cells to recipient cells in various mutant backgrounds via *kar*-mediated transfer (see below). Clones containing the correct phenotypes were confirmed by PCR with primers M13–20 and M13Rev (all primers used in this study can be found in Supplementary Table 1), followed by direct DNA sequencing.

### YAC transfer (*kar*-cross)

Donor cells 213 cir (canavanine-sensitive) containing YACs with the Z-DNA or control B-DNA sequences were grown from a single colony in minimum synthetic defined (SD) base with CSM-Leucine-Uracil media (MP Biomedicals, Santa Ana, CA) overnight at 30 °C. A single colony of BY4742 canavanine-resistant recipient cells from the yeast deletion library was grown in yeast extract peptone dextrose media (YPD) at the permissive temperature overnight of 27–30 °C. YACs were transferred from donor cells to recipient cells via kar-mediated transfer^51^. Briefly, equal optical density (OD) of donor cells with YACs and recipient mutant cells were mixed and inoculated into fresh YPD media, and grown in a shaking incubator for 6 h at the permissive temperature. The culture was plated on CSM-Ura-Arg + canavanine (100 mg L^−1^) plates, and incubated at the permissive temperature. *Kar*-crossed cells were plated for single colonies on CSM-Leu-Ura plates containing canavanine and CSM-lys to confirm the transfer (i.e., colonies containing the YAC will grow on CSM-Ura-Leu, but not on CSM-Lys plates). Cells were analyzed via direct DNA sequencing using the M13–20 primer (flanking the insert) to confirm the presence of the YAC and the correct Z-DNA-forming or control B-DNA-forming sequence.

### YAC fragility assay

Five single clones containing YACs with either the Z-DNA-forming or control B-DNA-forming sequence were used to inoculate 2 mL cultures in SD base with CSM-Leu media, and were grown for 20 h at a permissive temperature of 27–30 °C. For each culture, 50 μL was plated on SD base with CSM-Leu plates containing 5-fluoroorotic acid (5-FOA) (Zymo Research, Irvine, CA) to select for mutagenic events resulting in the loss of functional *URA3* on the YAC. Each culture was diluted 1:10,000 and plated on SD base with CSM-Leu plates to serve as a total cell number count. All colonies that grew on CSM-Leu + 5-FOA and CSM-Leu plates were counted for the experiment. The mutation rates were calculated using the Ma-Sandri-Sarkar Maximum Likelihood Estimator (MSS-MLE) Method of the Fluctuation AnaLysis CalculatOR (FALCOR)^52^. This assay was performed in triplicate on five separate clones (3 cultures for each clone) for the Z-DNA-containing and control YACs for all yeast strains analyzed in this study. Student’s *t*-test was used to determine significant differences of the averages between strains using the average of the triplicate of the representative clone.

### YAC mutation spectra analysis

For each strain containing the Z-DNA or control B-DNA YAC studied in the fragility assay, 30 FOA^R^ colonies were inoculated in water and lysed by boiling at 95 °C for 5 min, and used as template for PCR with the sets of primers listed in Supplementary Table 1. The M13 primer set was specific to the Z-DNA-forming or control B-DNA-forming region, the URA set serving as a measure of FOA^R^, and the LEU set served as a loading control. Results obtained from PCR and gel electrophoresis were categorized according to potential causes of FOA^R^ as follows: (1) samples with amplification from only the LEU primer set were categorized as having a double-strand break (DSB) and loss of the right arm of the YAC; and (2) all other samples with amplification from all three sets of primers or a combination of any two were categorized as having a point mutation or small deletion within the *URA3* gene. Amplified DNA was separated on a 1% agarose gel, stained with ethidium bromide, and visualized on a ChemiDoc Imaging System (Bio-Rad, Hercules, CA).

### Z-DNA-induced mutagenesis assay in human cells

Human XPA-deficient (XPA12RO) and XPA-proficient [XPA12RO clone 12 (SV40-immortalized human XPA fibroblasts complemented with the XPA gene)] cells were obtained from Dr. Richard Wood (University of Texas MD Anderson Cancer Center, Science Park Research Division), and were cultured as described^53^. Isogenic human XPF-proficient (GM08437B-XPF) and XPF-deficient cells (GM08437B-pLPC)^54^, and siRNA-depleted MSH2 cells were maintained in Dulbecco’s Modified Eagle Medium (DMEM, Life Technologies, Carlsbad, CA) with 10% fetal bovine serum (FBS) and antibiotics. A Z-DNA (pUCG14) or control (pUCON) plasmid was transfected into human cells using GenePORTER (Genlantis Inc., San Diego, CA) according to the manufacturer’s recommendations using 3 μg of plasmid DNA. Cells were harvested 48 h following transfection, and plasmid DNA was extracted using Hirt’s method^19^.

Purified plasmid DNA was digested with DpnI to remove plasmids that were not replicated in the mammalian cells, and again purified using phenol-chloroform extraction and ethanol precipitation prior to transformation via electroporation into *E. coli* DH10β electrocompetent bacterial cells (New England Biolabs, Inc., Ipswich, MA), which were used in the *lacZ*-based blue-white screening assay to determine mutation frequencies^19^. Following electroporation, liquid cultures were grown at 37 °C for 1 h, at which time various dilutions were plated onto LB agar containing IPTG + X-Gal and grown at 37 °C for 24 h to screen for mutants (white colonies *lacZ*^*-*^) as a frequency compared to wild-type (blue colonies, *lacZ*^*+*^). Experiments were performed in triplicate and Student’s *t*-test was used to calculate statistical values. DNA from individual mutant colonies was sequenced to characterize the mutations/deletions near the Z-DNA or control B-DNA regions.

### Depletion of MSH2 in human cells by siRNA knockdown

Human MSH2 siRNA or non-targeting siRNA (Dharmacon, GE Healthcare) (Supplementary Table 1) was transfected into cultured human XPF-proficient cells (GM08437B-XPF) with GenePORTER (Genlantis Inc., San Diego, CA) using the manufacturer’s recommended protocol at a final siRNA concentration of 0.1 μM. A second siRNA transfection, together with the pUCG14 and pUCON mutation-reporter plasmids, was carried out 48 h (T_48_) after the first transfection using GenePORTER at a final siRNA concentration of 0.1 μM and 3 μg of plasmid DNA. Cells were harvested at T_48_ as well as 16 or 48 h after the second transfection (T_64_ and T_96_) as indicated for Western blotting (described below) to confirm the knockdown of MSH2 (Supplementary Fig. 3), and plasmids were extracted for analysis by mutagenesis and ChIP assays.

### Determination of siRNA knockdown by Western blotting

Human XPF-proficient cells (GM08437B-XPF) were harvested 48 h after plasmid transfection. Cells were lysed in 1x radioimmunoprecipitation assay (RIPA) buffer (with proteinase inhibitor cocktail) on ice for 1 h, followed by sonication. A portion of the supernatant (equivalent to 10–30 μg of protein) was mixed with 2x SDS loading buffer and boiled for 5 min at 95 °C. Samples were separated by SDS-PAGE on a 4–15% Criterion™ TGX™ (Tris-Glycine-eXtended) midi gel, and transferred to a polvinylidene difluoride (PVDF) membrane using a Trans-Blot® Turbo™ Transfer System (Bio-Rad, Hercules, CA). The membrane was blocked for 1 h at room temperature with 1x TBST [tris buffered saline (0.02 M tris base, 0.15 M NaCl) plus 0.05% Tween 20] with 5% blotting grade dry milk (Bio-Rad), and probed with antibodies against MSH2 (1:100, Calbiochem, Billerica, MA, #PC57), XPF (1:1000, Cell Signaling, #13465 s), or PCNA (1:10,000, Santa Cruz, #sc-56), and visualized on a ChemiDoc Imaging System. ImageJ software was used to quantify band intensity.

### Chromatin immunoprecipitation assay in human cells

Detection of protein enrichment at a Z-DNA-forming site was performed as previously described with some modifications^55^. Human cells were plated in antibiotic-free Dulbecco’s Modified Eagle Medium (DMEM, Life Technologies, Carlsbad, CA) with 10% fetal bovine serum (FBS) at a concentration of 240,000 cells/plate in 60 mm plates. The next day, 3 μg of pUCG14 or pUCON was transfected into the cells using GenePORTER (Genlantis). At 16 h post-transfection, the cells were fixed with formaldehyde at a final concentration of 1% for 10 min at room temperature. The remainder of the ChIP assay was performed using the Simple ChIP Enzymatic Chromatin IP Kit (Cell Signaling, Inc., Santa Cruz, CA), following the manufacturer’s protocol with some modifications. Aliquots of the chromatin were incubated with 1 μg of primary antibody [α-IgG and α-H3 antibodies (ChIP kit), α-MSH3 antibody (Thermo Fisher, #PA529829, 0.04 μL μL^−1^ reaction), α-MSH2 antibody (Calbiochem, #PC57, 0.02 μL μL^−1^ reaction), α-MSH6 antibody (Abcam, #ab14204, 0.01 μL μL^−1^ reaction), α-XPA antibody (Abcam, #ab85914, 0.01 μL μL^−1^ reaction), and α-XPF antibody (generous gift from Dr. Richard Wood, University of Texas M.D. Anderson Cancer Center, 0.01 μg μL^−1^ reaction) at 4 °C with rotation overnight. The remainder of the assay was performed following the ChIP kit protocol. DNA fragments were purified using the Wizard® SV Gel and PCR Clean-Up System (Promega, Madison, WI). Fractions of purified ChIP and input DNA were used for PCR analysis. Primers used for PCR amplification included pUinsFor1 and pUinsRev1 (Supplementary Table 1). Amplified products were separated on a 1% agarose gel, stained with ethidium bromide, and visualized on a ChemiDoc Imaging System. ImageJ software was used to quantify band intensity. Experiments were performed in triplicate and Students’ *t*-test was used to calculate statistical values.

### S1 nuclease sensitivity assays

As previously described^1^ with slight variations, Z-DNA-forming or control B-DNA-forming plasmids (2 μg) (pUCG14 and pUCON, respectively), were incubated at room temperature for 5 min with 50 mM NaCl and 4 mM MgCl_2_, then digested with 2 μL S1 nuclease (Promega Corporation, Madison, WI) in 25 μL reactions with provided S1 reaction buffer for 20 min at 37 °C. Reactions were stopped by heat inactivation at 65 °C for 20 min and diluted to 100 μL. Plasmid DNA was extracted using phenol chloroform, and precipitated using ethanol. If indicated, plasmid DNA was furthered digested with a combination of 1 μL each of NdeI (at position 720) and/or AflII (position 1716) endonucleases in 20 μL reactions for 3 h at 37 °C. Reactions were stopped by heat inactivation at 65 °C for 20 min. Cleaved products were separated on a 1.5% agarose gel, at 50 volts for 8 h, stained with SYBR® Gold (Life Technologies, Carlsbad, CA), and visualized on a ChemiDoc Imaging System. Experiments were performed in triplicate.

### In vitro DNA cleavage assay via primer extension

Isogenic human XPF-proficient (or XPF-deficient WCE were prepared from human XPF-proficient or deficient cells (described above) using the Nucbuster™ Protein Extraction Kit (EMD Millipore, Temecula, CA). Reaction mixtures containing 600 ng of pUCG14 or pUCON were incubated at room temperature for 5 min with 50 mM NaCl and 4 mM MgCl_2_. Samples were then incubated in reaction buffer (5 mM MgCl_2_, 40 mM Hepes-KOH pH 7.8, 0.5 mM DTT, 2 mM ATP, 22 mM phosphocreatine, 0.36 mg mL^−1^ BSA, 50 ng μL^−1^ CPK, 30 mM KCl), and water or 100 ng of WCE for 30 min at 30 °C. After incubation, samples were treated with 0.02 M ethylenediaminetetraacetic acid (EDTA) and 80 μg mL^−1^ RNAse A and further incubated at 37 °C for 10 min. Samples were then treated with 0.5% SDS and 1 mg mL^−1^ proteinase K and incubated at 65 °C for 30 min. Plasmid DNA was purified using phenol-chloroform and ethanol precipitation, resuspended in nuclease-free water and used as template for PCR with JMLeft or JMRight primer to detect nicks on the plasmid DNA (Supplementary Table 1). Amplified DNA was separated on a 1.5% agarose gel, stained with SYBR® Gold (Thermo Fisher Scientific, Waltham, MA) and visualized on a ChemiDoc Imaging System (Bio-Rad, Hercules, CA). Experiments were performed in triplicate.

### Modeling and docking of protein-DNA complexes

The DNA and protein structures were downloaded from the Protein Data Bank (PDB). A model of the ERCC1-XPF complex was generated using the program PyMol [http://www.pymol.org]. The protein complex was constructed using Swiss-Model from the XPF protein [https://www.uniprot.org/uniprot/Q92889] with the XPF HhH domains [http://www.rcsb.org, 10.2210/pdb1z00/pdb]^56^ and the nuclease domain of human XPF bound to dsDNA as a template [http://www.rcsb.org, 10.2210/pdb2bgw/pdb]^56^. The DNA excision repair protein ERCC1 was modeled [https://www.uniprot.org/uniprot/P07992] with the ERCC1 central domain template [http://www.rcsb.org, 10.2210/pdb2jpd/pdb]^57^. The final complex was generated after protein-protein docking of ERCC1 with XPF using Z-Dock^58^ [http://zdock.umassmed.edu]. A model of the MSH2-MSH3 complex was generated using the program PyMol [http://www.pymol.org]. The crystal structures of the MSH2 and MSH3 proteins were downloaded as a complex from the Protein Data Bank [http://www.rcsb.org, 10.2210/pdb3thy/pdb]^30^. The crystal structure of Z-DNA [http://www.rcsb.org, 10.2210/pdb2acj/pdb]^59^ along with the B-DNA, highlighting the B-Z junction, were also downloaded from PDB.

We used both NPDock [http://www.genesilico.pl/NPDock] and HDock software [http://hdock.phys.hust.edu.cn] to carry out the docking studies between the ERCC1-XPF or MSH2-MSH3 complexes and the Z-DNA structure. NPDock combines GRAMM for global macromolecular docking^29^, scoring with a statistical potential, clustering of best-scored structures, and local refinement. HDock uses hybrid algorithms for docking and refining structures.

We used the Schrödinger maestro (Release2019-2) and Pymol program [https://www.schrodinger.com/] to model, visualize, and represent the proteins and Z-DNA. It was also used to identify the interacting residues, measure the distances between atoms, and model the interface residues. Residue name and number of the interacting partners were also highlighted using this software.

### Statistics

Most data are presented as bars with means ± SEM, and values of each repeat are shown as dot plots on the bars. For comparison of two groups, *P* values refer to unpaired, two-tailed Student’s *t*-test. *P* < 0.05 was considered statistically significant. The tests used for each experiment (e.g. Students’ *t*-test), and times of replicates (at least three independent repeats) are described in the Methods section and in the Figure Legends.

### Reporting summary

Further information on research design is available in the Nature Research Reporting Summary linked to this article.

## Supplementary information

Supplementary Information

Reporting Summary

## Data Availability

All relevant data needed to evaluate the conclusions are available within the article and Supplementary Information. The link to the source data of protein and DNA structures in Fig. 4 are provided in the Methods section. All other relevant data are provided in the “source data file”, and available from the corresponding author on request.

## Source data

Source Data
