# Supplementary material for: Distinct DNA repair pathways cause genomic instability at alternative DNA structures
Source: Nat Commun. 2020 Jan 13;11:236. doi: 10.1038/s41467-019-13878-9 (PMC6957503; doi:10.1038/s41467-019-13878-9)
Supplement: Supplementary file 1 — Supplementary Information [file 41467_2019_13878_MOESM1_ESM.pdf]

Supplementary Information

Distinct DNA Repair Pathways Cause Genomic Instability at Alternative DNA Structures

J.A. McKinney, G. Wang et al.

Supplementary Methods

Quantitative polymerase chain reaction (qPCR). qPCR was performed using a ViiA 7 q-PCR system (Applied Biosystems, Foster City, CA) to determine the Ct values from the ChIP samples. All qPCR reactions were performed in triplicate using the iTaq Universal SYBR Green supermix (BIO-RAD, Hercules, CA) as per the manufacturer's suggested protocol. Specific primers were designed to detect a control B-DNA (pUCON) region, a Z-DNA-forming region (pUCG14), a G4-forming region (pMyc-TT), or a region 110-bp downstream of the Z-DNA sequence. The primer sequences are listed in Supplementary Table 1. The samples were denatured at 95°C for 20 seconds and annealed/amplified at 60°C for 30 seconds for 40 cycles. Real-time fluorescent data of FAM and VIC were collected and analyzed using the ViiA 7 Software v1.2.3. Data were analyzed as per the instructions on the ThermoFisher Scientific website [https://www.thermofisher.com/us/en/home/life-science/epigenetics-noncoding-rna-research/chromatin-remodeling/chromatin-immunoprecipitation-chip/chip-analysis.html]. Anti-DNA-RNA Hybrid, clone S9.6 (Cat. No. MABE1095) antibody was used at a 1:50 dilution. Anti-MSH2 antibody (Calbiochem) and anti-XPF antibody (generous gift from Dr. Richard Wood, University of Texas M.D. Anderson Cancer Center) were used at a 1:1000 dilution (1 μg per 1000 μL).

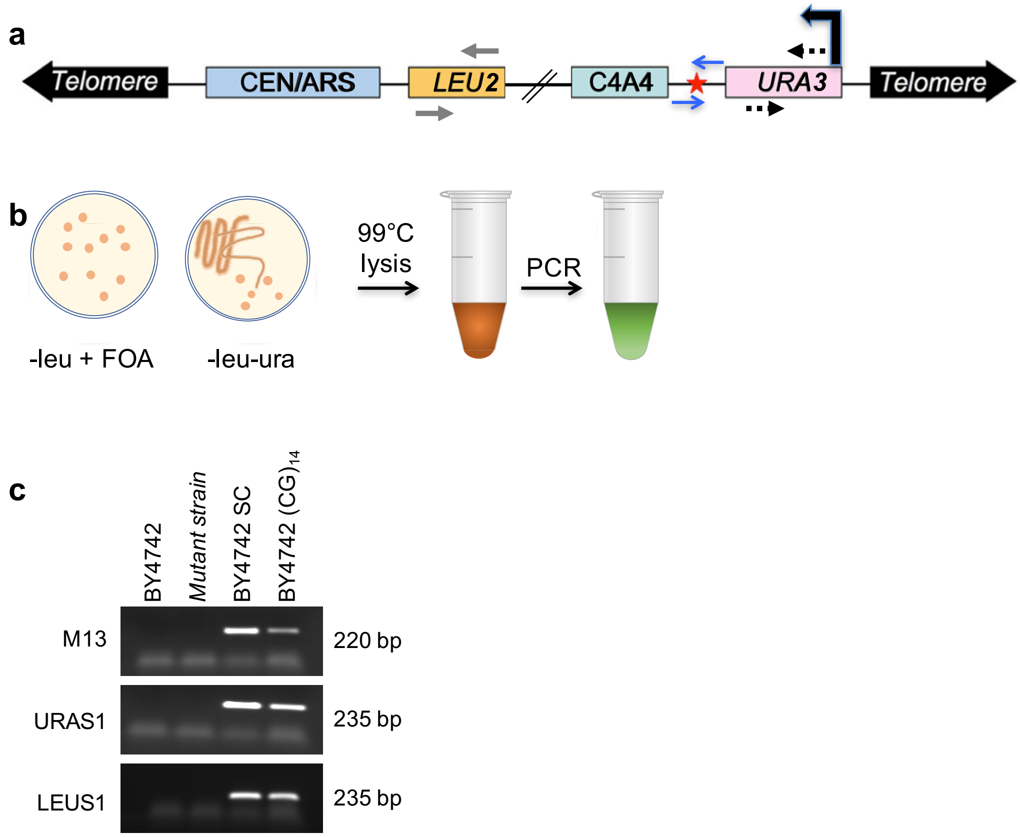

Supplementary Figure 1. Characterization of mutations on YACs in *S. cerevisiae*. a, To determine the type of mutation that occurred in the *URA*3 gene to result in FOA^R, several primer sets were designed that were specific to the *URA3* gene (black dashed arrows), Z-DNA or control B-DNA insert (blue arrows), or the distal region in the *LEU2* gene, which also served as a loading control (dark grey arrows). The black arrow represents the direction of the *URA3* promoter. **b,** To determine the mutation spectra, 30 single colonies from the FOA-containing or CSM-leu-ura plates were harvested, lysed at 99°C for 5 minutes, and used as templates for the PCR reactions. Arm loss was characterized by those colonies in which only the distal *LEU* product was detected, but where the Z-DNA segment and the *URA3* gene were lost in the PCR amplification. If the Z-DNA segment or the *URA3* gene was detected by PCR, then the inactivation of the *URA3* gene was from an inactivating point mutation within the *URA3* gene and was defined as a point mutation (PM). **c,** BY4742 and a mutant strain that did not contain the YAC were used to verify that the primers did not amplify regions in the yeast genome, and were specific to the YAC. BY4742 SC contains the control B-DNA-forming YAC, and BY4742 CG14 contains the Z-DNA-forming YAC. The lighter bands on the gel are primer dimers.

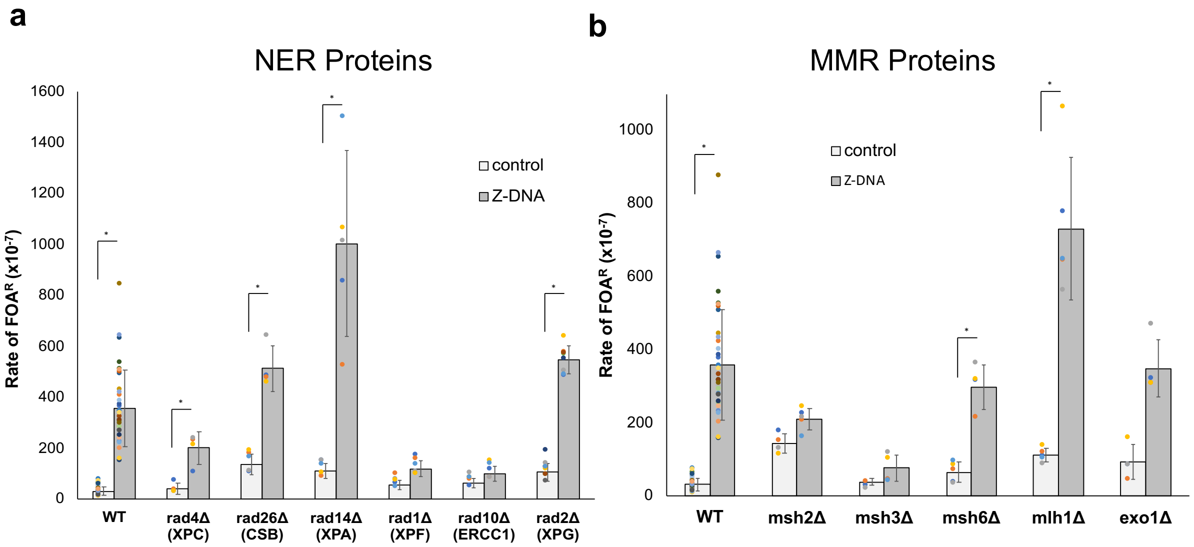

Supplementary Figure 2. Mutation rates of Z-DNA-forming YACs in *S. cerevisiae.* The YAC fragility assay was performed on BY4742 wild-type (WT) cells, mutant strains deficient in **a,** NER, or **b,** MMR proteins containing either a control B-DNA-forming YAC (control) or a Z-DNA-forming YAC (Z-DNA) as measured by rate of FOA^R. [Student's *t*-test was used to calculate *P* value. * = P<0.001]. Data are presented as means ± SEM of independent repeats, and values of each repeat are shown as dot plots on the bars.

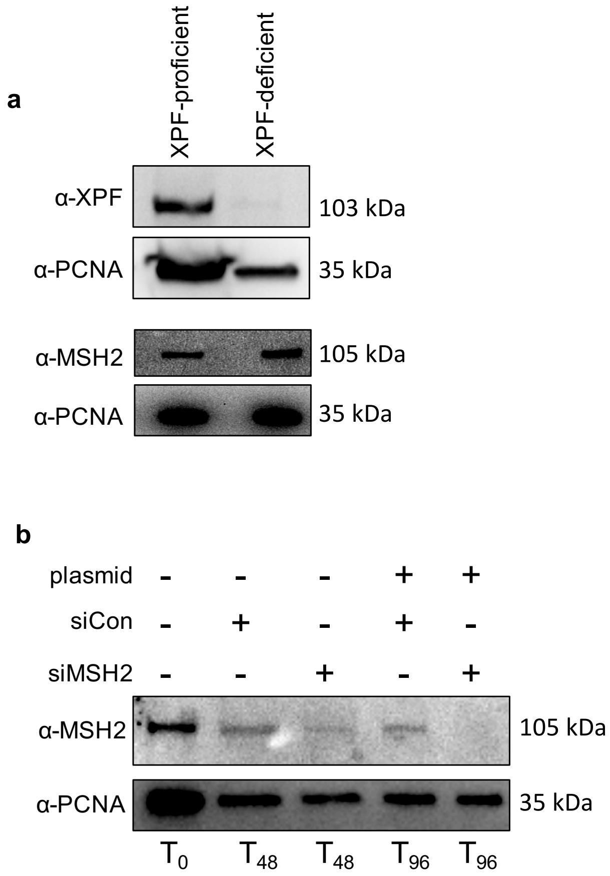

Supplementary Figure 3. Human cell lines used for mutagenesis assays. a, Human XPF-proficient and XPF-deficient cell lines were confirmed by Western blotting using antibodies against XPF (top panel). PCNA was used as a loading control. **b,** MSH2 depletion by siRNA knockdown in human XPF-proficient cells confirmed by Western blotting using an antibody against MSH2, with PCNA used as a loading control (bottom panel). Plasmid only and a non-targeting siRNA (siCon) were used as negative controls. Various time points (T) were tested, indicating hours following the first transfection.

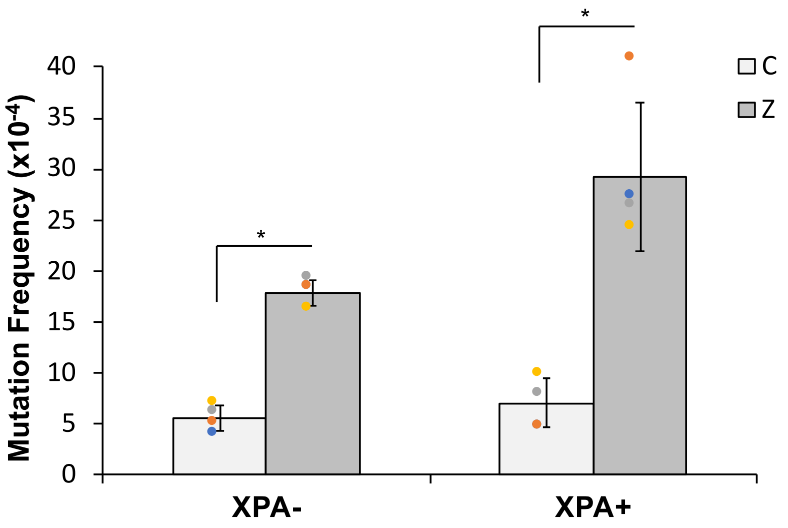

Supplementary Figure 4. XPA is not required for Z-DNA-induced mutagenesis. Blue-white mutation assays were performed on isogenic WT and XPA-deficient human cells using plasmids containing control B-DNA (C) or Z-DNA-forming sequences (Z). [Student's *t*-test was used to calculate *P* value. * = P<0.05]. Data are presented as means ± SEM of independent repeats, and values of each repeat are shown as dot plots on the bars.

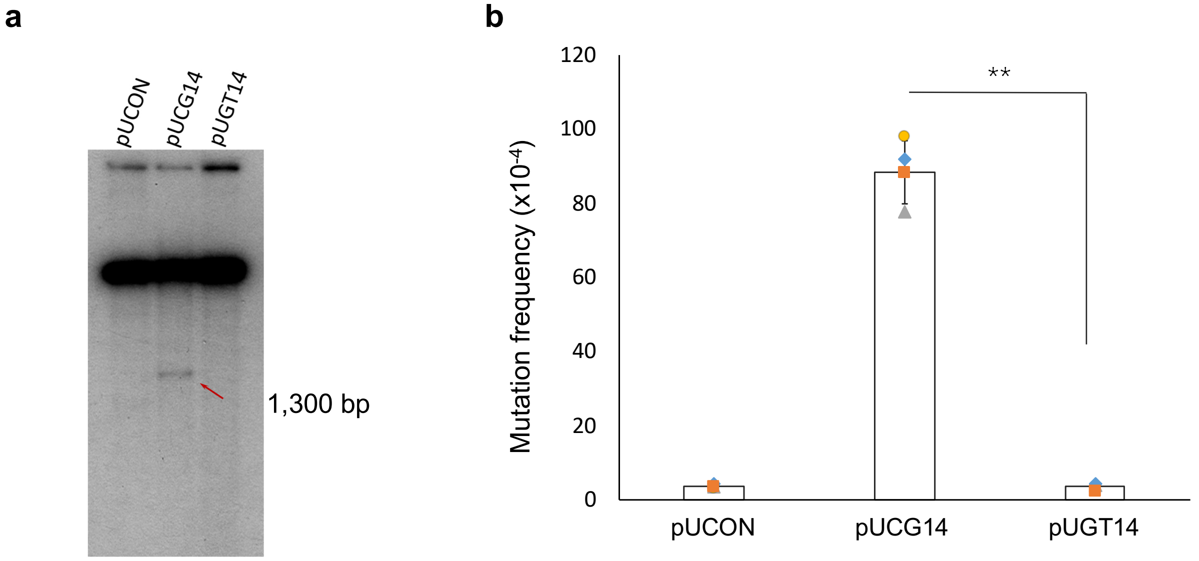

Supplementary Figure 5. Short repeats unable to adopt Z-DNA structures are stable. a, Identification of the Z-DNA conformation at the insertion site. The insert-containing plasmids were treated with single-strand DNA specific S1 nuclease and the breakpoints were radiolabeled. Restriction digestion released an ~3,000 bp fragment, and S1 digestion at the ssDNA formed at the B-Z junctions created a shorter ~1,300 bp fragment, as indicated by the red arrow. **b,** A GT14 repeat that cannot form Z-DNA is not mutagenic in mammalian COS-7 cells compared to Z-DNA forming CG14 repeat. Blue-white mutation assays were performed using *lacZ* mutation-reporter vectors. [Student's *t*-test was used to calculate *P* values < 0.01 (**)]. Blue-white mutation assays were performed using *supF* mutation-reporter vectors. [Student's *t*-test was used to calculate *P* values. ** = P<0.01]. Data are presented as means ± SEM of independent repeats, and values of each repeat are shown as dot plots on the bars.

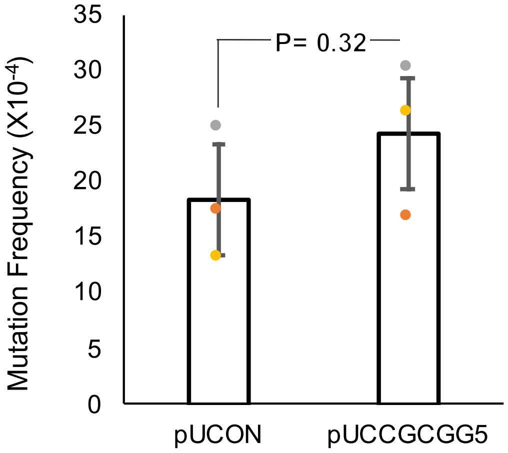

Supplementary Figure 6. Z-DNA conformation versus than a high CG content. A CCGCGG(5) repeat containing a similar CG content as the Z-DNA-forming CG14 repeat, but unable to adopt a Z-DNA structure, is not mutagenic in mammalian COS-7 cells. Blue-white mutation assays were performed using *lacZ* mutation-reporter vectors. [Student's *t*-test was used to calculate *P* values > 0.05]. Data are presented as means ± SEM of independent repeats, and values of each repeat are shown as dot plots on the bars.

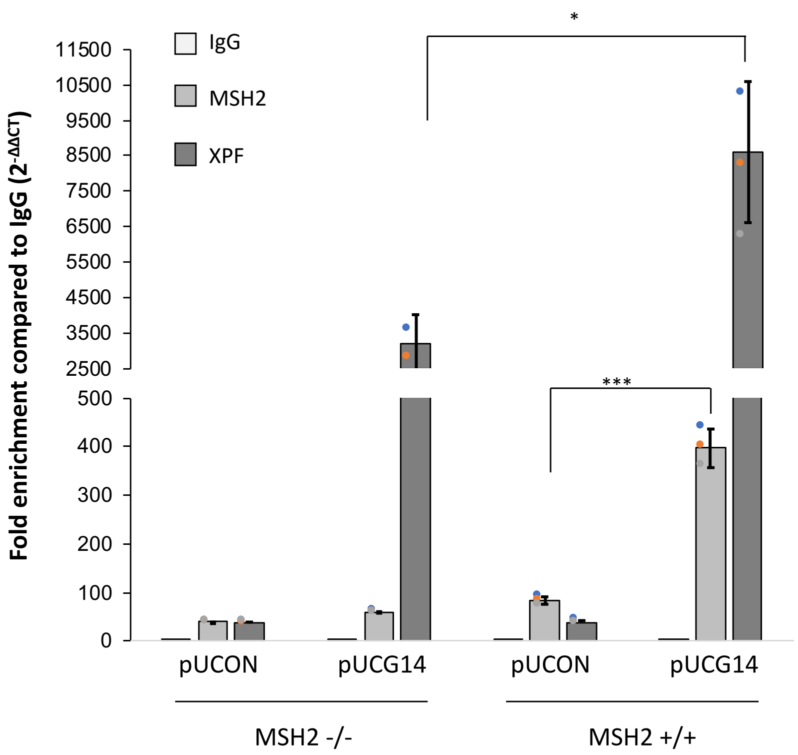

Supplementary Figure 7. MSH2 and XPF are associated with the Z-DNA-forming sequence. Plasmids with a control B-DNA-forming sequence (pUCON) or a Z-DNA-forming sequence (pUCG14) were transfected into human MSH2-deficient Hec59 cells or MSH2-expressing Hec59 cells (Hec59+Chr2). ChIP assays followed by qPCR analysis indicated a significant ~6-fold enrichment of MSH2 on the Z-DNA-forming sequence in MSH2-proficient (Hec59+Chr2) cells. Similar analysis indicated that XPF was significantly (~3-fold) enriched on the Z-DNA-forming sequence in the MSH2-proficient cells (Hec59+Chr2) when compared to MSH2-deficient cells (Hec59), suggesting a role of MSH2 in XPF recruitment to the Z-DNA-forming region. The fold-enrichment was calculated by comparing the ΔΔCT values of MSH2 and XPF against IgG (specificity control). The data plotted is an average of at least three independent experiments and each sample was amplified 3 times per experiment using qPCR. The error bars represent SD. *P* values were determined by nonparametric Mann-Whitney U test. A *P* value of less than 0.05 was considered significant. *** = $P<0.0001$. Values of each repeat are shown as dot plots on the bars.

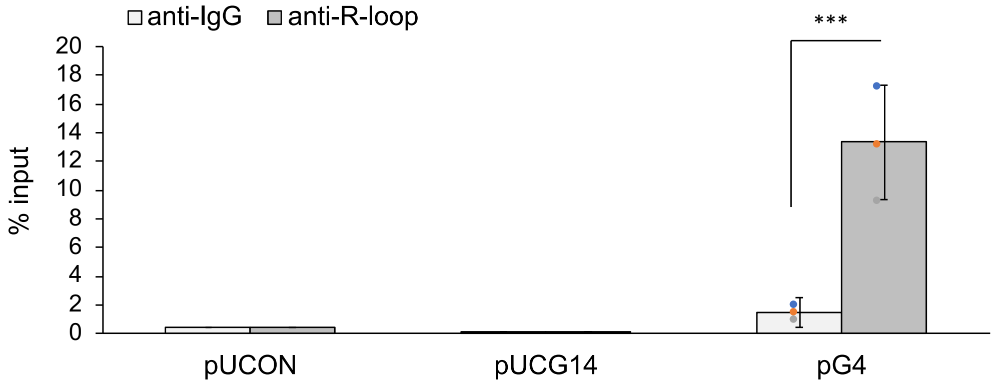

Supplementary Figure 8. The CG14 repeat does not form detectable R-loop structures. qPCR amplification of the Z-DNA-forming sequence (pUCG14) from ChIP assays in mammalian COS-7 cells shows no detectable enrichment when immuno-precipitated with an anti-R-loop antibody compared to the control B-DNA, while a G4-forming sequence used as a positive control showed an ~9-fold enrichment compared to the control sequences. The average CT values from three separate experiments were used to determine the % input values according to the manufacturer's suggested protocol (ThermoFisher). *P* values were determined by using a nonparametric Mann-Whitney U test. A *P* value of less than 0.05 was considered significant. *** = $P<0.0001$. Data are presented as means ± SEM of independent repeats, and values of each repeat are shown as dot plots on the bars.

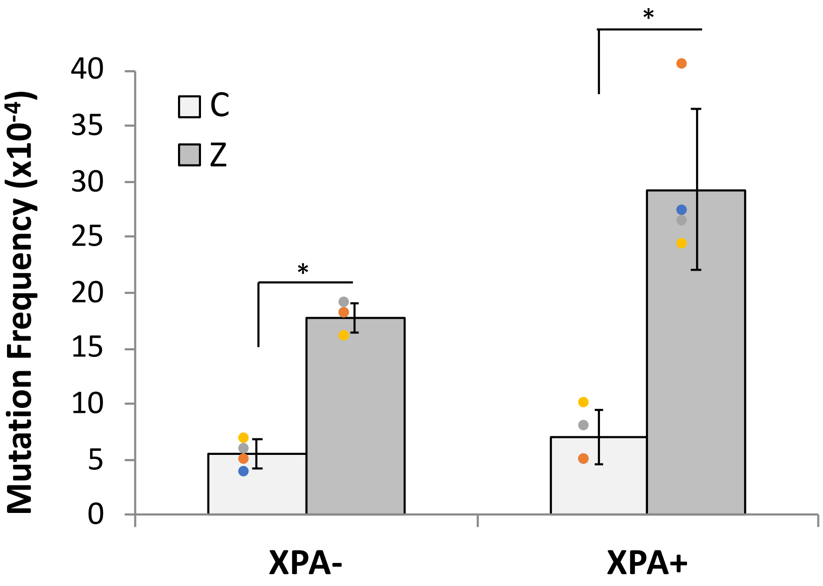

Supplementary Figure 9. SAW1 deficiency does not affect Z-DNA-induced fragility in *S. cerevisiae.* YAC fragility (loss of the *URA3* gene) induced by Z-DNA (Z) and a control B-DNA sequence (B), was measured as FOA^R in WT and *saw1*Δ strains [Student's *t*-test was used to calculate P value. * = P<0.001]. Z-DNA induced ~5x fold more fragility over control in both WT and *saw1*Δ strains. Data are presented as means ± SEM of independent repeats, and values of each repeat are shown as dot plots on the bars.

Supplementary Table 1 List of oligonucleotides used in this study

Region	Name	Sequence
B-DNA insert	control sequence	CGAGCTATCTGAGTCGAATACAGTTCGAC
Z-DNA insert	ZFS[a]	CGCGCGCGCGCGCGCGCGCGCGCGCGCG
CCGCGG5 insert	CCGCGG5	CCGCGGCCGCGGCCGCGGCCGCGGCCGCGG
AT14 insert	AT14	ATATATATATATATATATATATATATAT
B-DNA insert	control sequence	CGAGCTATCTGAGTCGAATACAGTTCGAC
G4-DNA insert	G4	TCGAGCCCACCCCATAAGCGCCCCACCCTAGCG
Z-DNA and control on YAC[b]	M13-20	5' TGT AAA ACG ACG GCC AGT 3'
	M13Rev	5' CAG GAA ACA GCT ATG ACC 3'
URA3 gene on YAC[b]	Ura3S1For	5' AGG CGG CAG AAG AAG TAA CA 3'
	Ura3S1Rev	5' AAT GCG TCT CCC TTG TCA TC 3'
LEU2 gene on YAC[b]	Leu2S2For	5' CTG TGG GTG GTC CTA AAT GG 3'
	Leu2S2Rev	5' CCA TCA CCA TCG TCT TCC TT 3'
Z-DNA and control on plasmid[b]	pUinsFor1	5' GTT TTC CCA GTC ACG ACG TT 3'
	pUinsRev1	5' TTT ATG CTT CCG GCT CGT AT 3'
Z-DNA and control on plasmid[b]	JMleft	5' GGA GAA AAT ACC GCA TCA GG 3'
	JMright	5' ATT AGG CAC CCC AGG CTT TA 3'
G4 and control on plasmid[b]	G4left	5′-GCC CCC CTG ACG AGC ATC AC 3'
	G4right	5' TAG TTA CCG GAT AAG GCG CAG CGG 3'
110-bp downstream of Z-DNA[b]	RZ-1 left	5′-AAG TGT AAA GCC TGG GGT GC 3'
	RZ-1 right	5'-CGG ACT CAC CAT AGG GAC CA 3'
MSH2	MSH2 siRNA	U.C.U.G.C.A.G.A.G.U.G.U.U.G.U.G.C.U.U.U.U.

[a]Z-DNA-forming sequence
[b]Primer sequences

Supplementary Table 2 Mutation spectra of wild-type and repair-deficient yeast strains[a]

Strain	YAC	% PM del^{-1}	% arm loss (DSB)
		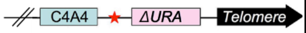	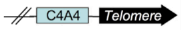
**WT[b] (BY4742)**	**C**	**60**	**40**
	**Z**	**13**	**87**
rad4Δ[c] *(XPC)*	C	76	24
	Z	100	0
rad26Δ (CSB)	C	51	49
	Z	0	100
rad14Δ[c] *(XPA)*	C	100	0
	Z	90	10
rad1Δ[c] *(XPF)*	C	100	0
	Z	100	0
rad2Δ[c] *(XPG)*	C	93	7
	Z	90	10
rad10Δ[c] *(ERCC1)*	C	93	7
	Z	93	7
msh2Δ[c]	C	90	10
	Z	80	20
msh3Δ[c]	C	100	0
	Z	97	3
msh6Δ	C	63	37
	Z	27	73
mlh1Δ	C	80	20
	Z	60	40
exo1Δ	C	74	26
	Z	0	100

[a]Depictions under colored titles indicate resulting YAC present in mutant colonies.
[b]Mutation spectra of wild-type strain.
[c]Mutants with spectra notably different than wild-type are denoted in red. n≥30.
